# Supplementary material for: Nanoformulation of Seaweed Eisenia bicyclis in Albumin Nanoparticles Targeting Cardiovascular Diseases: In Vitro and In Vivo Evaluation
Source: Mar Drugs. 2022 Sep 27;20(10):608. doi: 10.3390/md20100608 (PMC9605150; doi:10.3390/md20100608)
Supplement: Supplementary file 1 [file marinedrugs-20-00608-s001.zip › marinedrugs-1909858-supplementary.pdf]

6. Supplementary material

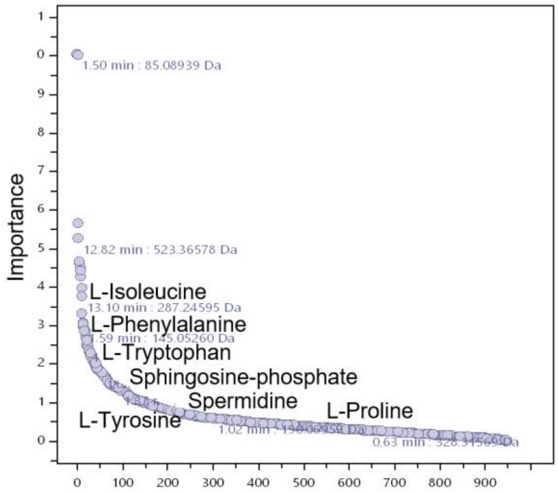

(**a**)

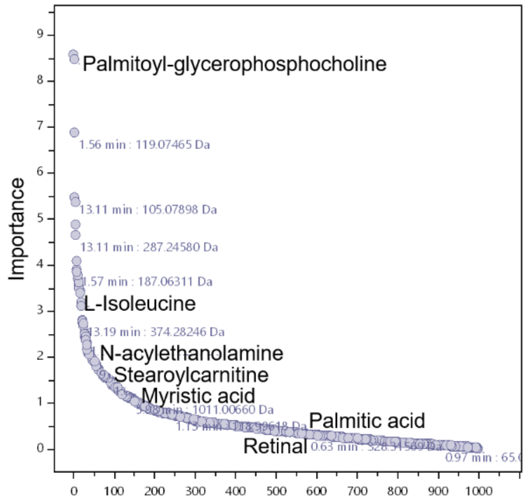

(**b**)

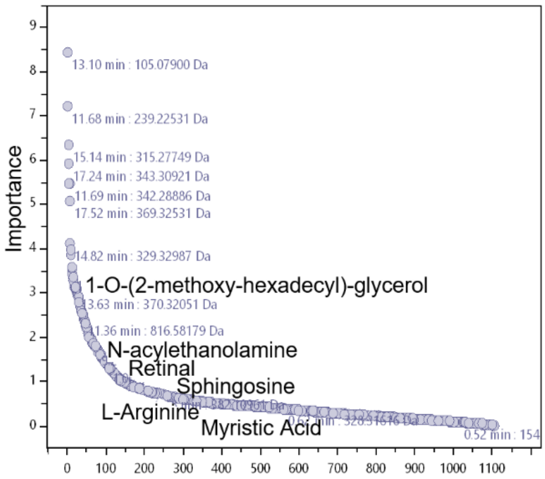

(**c**)

Figure S1. Projection on latent structures (PLS) analysis when (**a**) *Eisenia bicyclis* extract in the free form, (**b**) *Eisenia bicyclis* incorporated in BSA NPs and (**c**) Ezetimibe were administered. Results are from one rat of each test group that demonstrated the highest reduction in cholesterol level.

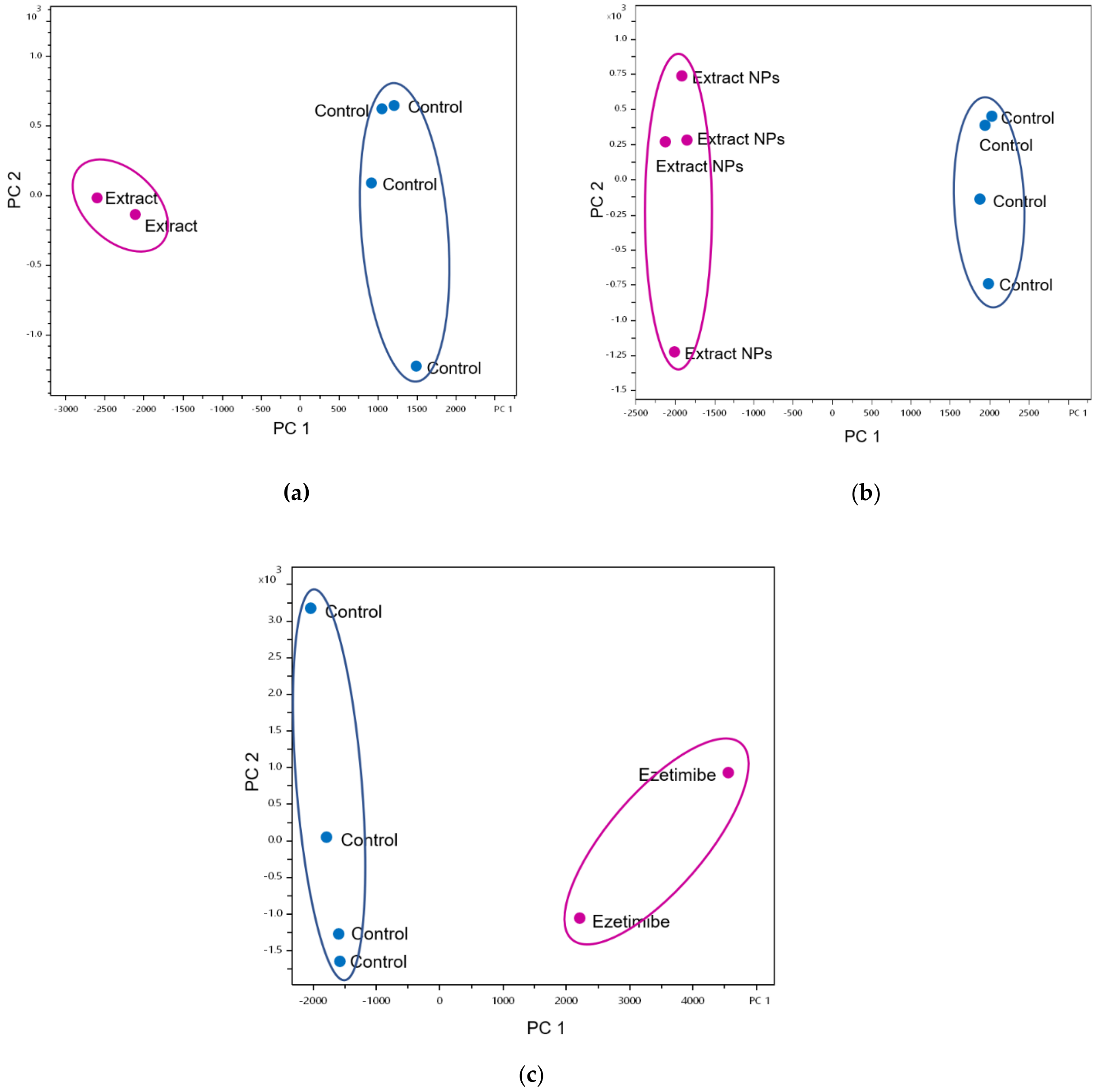

Figure S2. Principal Component Analysis (PCA) when (**a**) *Eisenia bicyclis* extract in the free form (Extract), (**b**) *Eisenia bicyclis* incorporated in BSA NPs (Extract NPs) and (**c**) Ezetimibe were administered. Results are from one rat of each test group that demonstrated the highest reduction in cholesterol level.

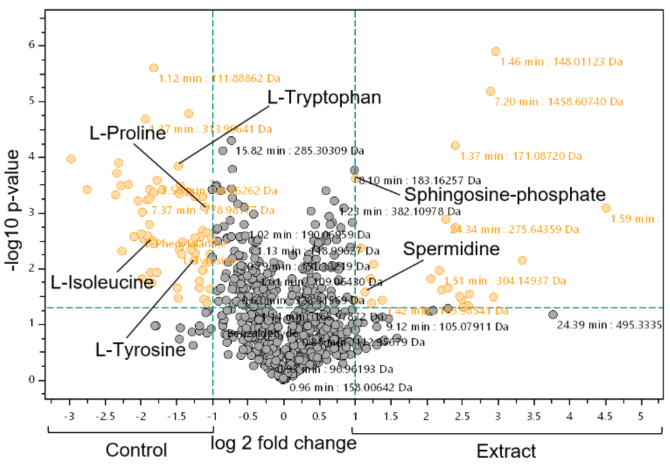

(**a**)

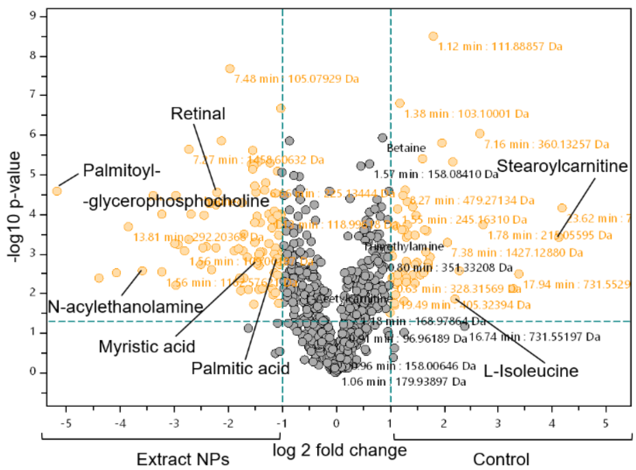

(**b**)

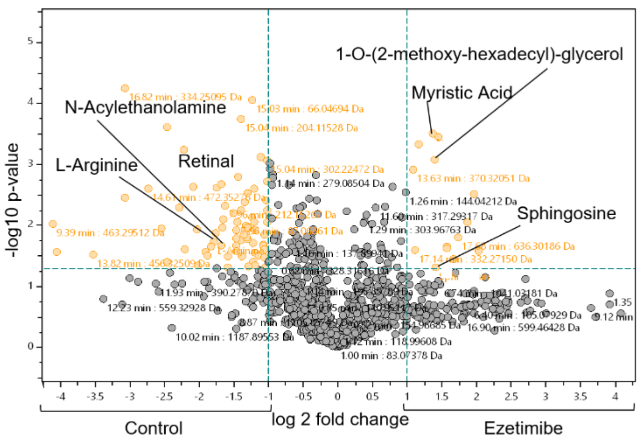

(**c**)

Figure S3. Volcano analysis when (**a**) *Eisenia bicyclis* extract in the free form, (**b**) *Eisenia bicyclis* incorporated in BSA NPs and (**c**) Ezetimibe were administered. Results are from one rat of each test group that demonstrated the highest reduction in cholesterol level.
